# The United States dried seahorse trade: A comparison of traditional Chinese medicine and ecommerce-curio markets using molecular identification

**DOI:** 10.1371/journal.pone.0291874

**Published:** 2023-10-03

**Authors:** J. T. Boehm, Eric Bovee, Stephen E. Harris, Kathryn Eddins, Ishmael Akahoho, Marcia Foster, Susan K. Pell, Michael J. Hickerson, George Amato, Rob DeSalle, John Waldman

**Affiliations:** 1 Sackler Institute for Comparative Genomics, American Museum of Natural History, New York, New York, United States of America; 2 Department of Biology, City College of New York, New York, New York, United States of America; 3 Subprogram in Ecology, Evolution Biology and Behavior, The Graduate Center of the City University of New York, New York, New York, United States of America; 4 School of Forest, Fisheries, and Geomatics Sciences, University of Florida, Gainesville, Florida, United States of America; 5 School of Natural and Social Science, SUNY Purchase College, Purchase, New York, United States of America; 6 The New School, New York, New York, United States of America; 7 Brooklyn Academy of Science and the Environment High School, Brooklyn, New York, United States of America; 8 Brooklyn Botanic Garden, Brooklyn, New York, United States of America; 9 Division of Invertebrate Zoology, American Museum of Natural History, New York, New York, United States of America; 10 Biology Department, Queens College, City University of New York, New York, United States of America; University of Veterinary Medicine Vienna: Veterinarmedizinische Universitat Wien, AUSTRIA

## Abstract

Tens of millions of dried seahorses (genus *Hippocampus*) are traded annually, and the pressure from this trade along with their life history traits (involved parental care and small migration distances and home ranges) has led to near global population declines. This and other forms of overexploitation have led to all seahorse species being listed in Appendix II under the Convention on International Trade in Endangered Species of Wild Fauna and Flora (CITES). The signatory nations of CITES recommended a 10-cm size limit of seahorses to ensure harvested individuals have reached reproductive maturity, and have thus had the chance to produce offspring, to maintain a more sustainable global seahorse fishery. We assessed adherence to CITES recommendations using DNA barcoding and size measurements to compare two prominent U.S. dried seahorse markets: (1) traditional Chinese medicine (TCM), and (2) non-medicinal ecommerce and coastal curio (ECC). We also estimated U.S. import abundance from CITES records. Of the nine species identified among all samples (n = 532), eight were found in the TCM trade (n = 168); composed mostly (75%) of the Indo-Pacific species *Hippocampus trimaculatus*, and *Hippocampus spinosissimus*, and the Latin American *Hippocampus ingens*. In contrast, ECC samples (n = 344) included 5 species, primarily juvenile Indo-Pacific *Hippocampus kuda* (51.5%) and the western Atlantic *Hippocampus zosterae* (40.7). The majority of TCM samples (85.7%) met the CITES size recommendation, in contrast to 4.8% of ECC samples. These results suggest non-size discriminatory bycatch is the most likely source of imported ECC specimens. In addition, CITES records indicate that approximately 602,275 dried specimens were imported into the U.S. from 2004–2020, but the exact species composition remains unknown as many U.S. imports records list one species or *Hippocampus* spp. from confiscated shipments due to difficulties in morphological identification and large numbers of individuals per shipment. Molecular identification was used to identify the species composition of confiscated shipment imports containing undesignated species, and similar to TCM, found *H*. *trimaculatus* and *H*. *spinosissimus* the most abundant. By combining DNA barcoding, size comparisons, and CITES database records, these results provide an important glimpse into the two primary dried U.S. seahorse end-markets, and may further inform the conservation status of several *Hippocampus* species.

## Introduction

Global fishery market overexploitation is a growing concern across nations, but the same is not true for non-food fisheries [1]. The growth of traditional medicine has increased the global demand and exploitation of non-food species including seahorses (genus *Hippocampus*) (seahorses) [2,3]. Seahorses are commonly traded for traditional Chinese medicine (TCM), aquarium pets, and as souvenirs and crafts in ecommerce and coastal curio shops (ECC) [4–6]. Historically, seahorses have been collected throughout the Indo-Pacific, but declines in regional catch numbers and an increased demand for seahorses has expanded fisheries worldwide [6–11]. Calculating the tonnage of seahorse bycatch from shrimp trawlers in Vietnam [9] estimated tens of millions of seahorses are traded each year in as many as 80 countries [12], and while trade of live animals has decreased, the dried seahorse trade is still largely unregulated [13]. Most of the seahorses enter the trade as incidental bycatch [9,14]. Unlike targeted or monitored fishing practices, bycatch is non-selective and leads to difficulties in accumulating data regarding both size of individuals collected and species-specific exploitation [7,11,15–18].

**Fig 1:**
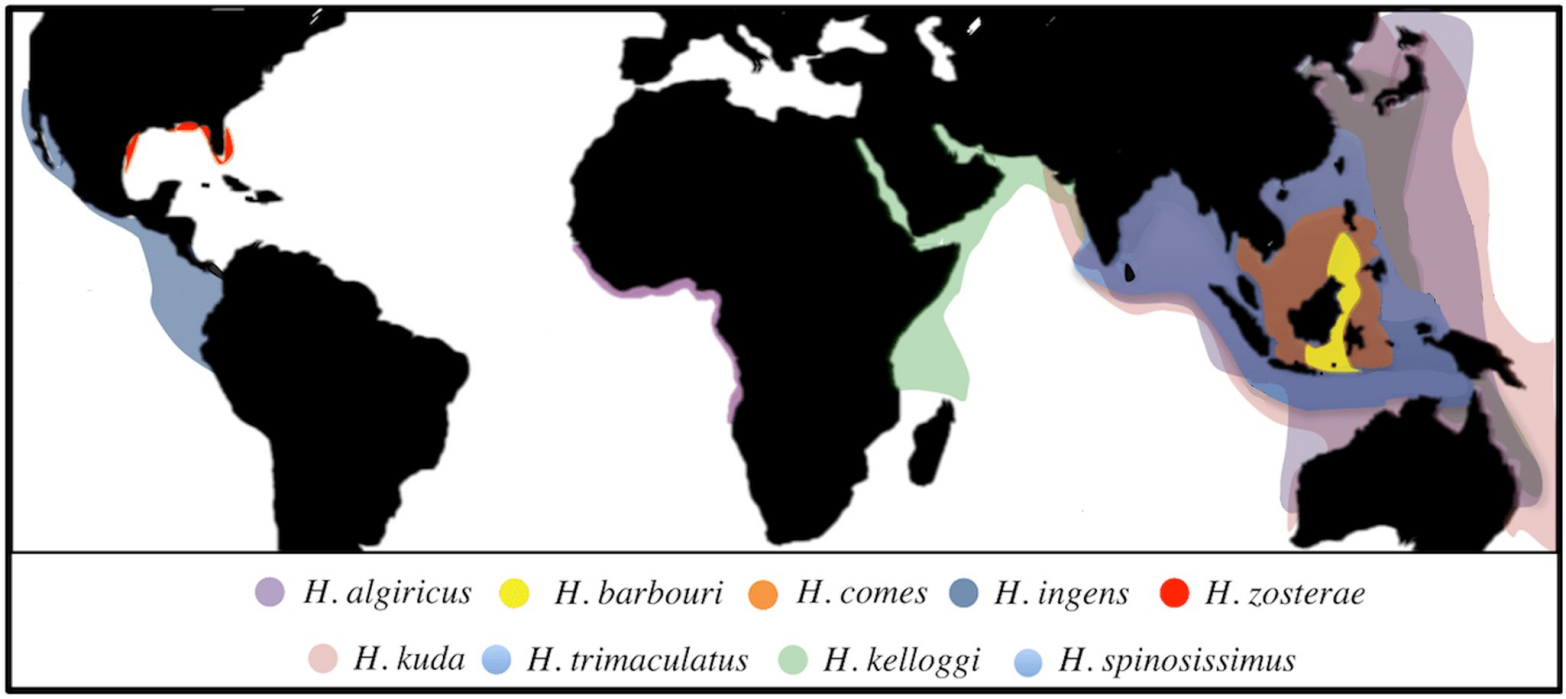
Distributions of seahorse species identified in this study. Distributions of each species identified in this study based on [5] and verified using data points derived from Ocean Biographic Information Systems (OBIS: www.iobis.org).

Seahorses are found in temperate and tropical nearshore environments globally (Fig 1). They make up only one genus (*Hippocampus)* in the family Syngnathidae with approximately 46 species [13]. Due to habitat loss and life history traits such as low active dispersal, low fecundity, sparse distribution, small home ranges, high parental investment, and male fidelity (which further reduces dispersal), many species are vulnerable to overexploitation [4,9]. These life history traits were found to be true across 32 seahorse species reviewed [4]. Across all species, densities were relatively low (0.5–10 individuals per square meter), lifespans were between one and five years, pairs are primarily monogamous with direct transfer of clutches to the male’s brood pouch, releasing anywhere between 5 and 2,000 young, and despite large variation in adult size, the size at sexual maturity across all species was below 7 cm [4]. Some of the large range of variation in adult size can lead to large differences in a species’ ability to respond to exploitation (See S1 Table for more detailed known life history traits of species identified in this study). Increased fishing pressure on seahorses prompted the Convention on International Trade in Endangered Species of Wild Fauna and Flora (CITES) to represent all species of *Hippocampus* on Appendix II [19]. This was an extraordinary step considering there are only a dozen marine fish groups represented out of approximately 35,000 CITES-listed species [12,14,20–24]. The CITES’ goal was to regulate exports that threaten wild populations, and there is evidence of some success in the reduction of live animals traded from wild populations [13], but the ban has done little to reduce the dried animal trade [25].

Additionally, as a bycatch-dominated fishery, the status of many seahorse populations is often unknown [17,24]. For example, of the 38 *Hippocampus* species listed on the International Union for the Conservation of Nature (IUCN) Red List of Threatened Species, 26 are considered “data deficient,” 10 as “Vulnerable,” one as “Endangered”, and one as “Least Concern”. All of the vulnerable species are exploited for trade, mostly as dried specimens which makes up ~98% of total trade [10,13]. More species are likely “vulnerable,” but current population trends remain “unknown” for 29 species and 19 evaluations “need updating” [26]. Therefore, the evaluation of seahorse species being imported into the U.S. market, or collected domestically, can inform efforts in seahorse conservation and increase our baseline knowledge of species, size, fishery pressures, and overall trade dynamics.

Trade records indicate that considerable numbers of live and dried seahorses are imported annually into the U.S. from Asia [6,25,27,28], with increasing exports to Asia for re-export from South America [8] and Africa [6]. Despite the total suspension of exports in some Asian countries in recent years, trade is still high with widespread lack of enforcement [29]. Traditional Chinese medicine in urbanized areas dominate the sale and purchase of dried seahorse specimens, yet in the U.S, dried seahorses are also purchased as curios for souvenirs and for crafts in coastal areas. We will use the term curio to represent all purchases for use as souvenirs or crafts. Ecommerce sites, including eBay and Etsy, allowed curio products to be easily purchased throughout the U.S. The growth in the seahorse curio market and its potential impact on seahorse populations led eBay to add dried seahorses to its “Animals and Wildlife Product Policy” that bans the sale in the U.S. of all seahorses. However, independent ecommerce sites can still sell curio products. In a recent search on eBay for ‘dried seahorses’, June 2023, 26 results actively advertised batches of dried seahorses for sale with 22 coming from the same seller in Algeria. This may get flagged before any sales are made, but it shows despite the ban, sellers still actively try to sell samples. In addition to the eBay policy and CITES listing, in 2004, the technical Animals Committee for CITES suggests that all signatory nations adhere to a 10-cm minimum size recommendation. Species identification is difficult among seahorses and because most species mature at a size less than 10 cm, the 10 cm limit should allow the majority of species to reach reproductive age before removal from the wild [8,19]. It is important to also evaluate specimen size to address at which developmental stages (i.e., juveniles or adults) particular species are being sold and in which end-market.

The use of DNA barcoding (identifying species from short homologous gene sequences) has become popularized since its development in 2003 [30,31] and is now a commonly utilized tool for species identification of animal products [32–34]. DNA barcoding is particularly useful for identification of species that are difficult to identify morphologically, like seahorses. To date, DNA barcoding has answered many questions including those involving the aquarium trade [35,36], fishery food traceability of morphologically unidentifiable specimen fragments such as shark fins and products [37–40], skate products [41], tuna species labeling [42,43], caviar source-species identification [44], and is now being used with high-throughput sequencing like Oxford Nanopore Technology’s (ONT) MinION for forensic genetic species identification [45]. DNA barcoding is also used in educational programs such as the Urban Barcoding Project (urbanbarcodeproject.org), as well as the development of high school and undergraduate curricula [46], and protocols have been developed to use miniaturized and portable equipment, like the MinION, by citizen scientists and natural resource professionals in the field [47,48]. Much of our work here was done in collaboration with local high school students working on independent research projects, and we hope that our provided curriculum (S1 File) contributes to this growing body of open source DNA barcoding protocols and provides insights to the illegal wildlife trade.

The primary focus of this study was to use DNA barcoding to identify and compare seahorse specimens from two dried seahorse markets in the continental USA: 1) traditional Chinese medicine (TCM) and 2) non-medicinal ecommerce and coastal curio (ECC) collected between 2009 and 2014. Species identification was based on two mitochondrial DNA markers that are commonly used for species identification: cytochrome b (cytb) and cytochrome oxidase subunit 1, (CO1) depending on which gene had the most sequences in NCBI for a given species. The nucleotide sequences of cytb and CO1 contain species-specific information and have been shown to readily amplify from dried seahorse specimens (cytb [8]) and across metazoans (CO1 [30]). Cytb use for seahorses is common, being used to investigate the wildlife trade [8], systematics [49–51] and population genetics [52–56]. In addition, using these markers, we determined the species composition of a confiscated shipment of seahorses from the Port of New York (2013) to determine if species matched those found in TCM or ECC markets. Both markets are often supplied by import shipments. and approximately 1/3 of all U.S. CITES import records lack species designations. This study focuses on using molecular identification of large batches often misidentified so live animals from the aquarium market were not included. Finally, we compared the size of specimens, and estimated the total number of individuals imported into the U.S from CITES records (2004–2020).

## Materials and methods

### Sampling design

To compare the species composition between TCM and ECC markets, 512 individuals were collected from 2009–2014. TCM samples were purchased from 13 stores (2–4 per area) from four Northeastern U.S. metropolitan “Chinatowns” (n = 168) (i.e., Philadelphia, PA; Brooklyn, NY; Queens, NY; and Manhattan, NY). ECC samples were purchased from 10 stores via eBay or independent websites (n = 300) as well as directly from four souvenir stores along the Texas Gulf coast (n = 44). See S2 Table for GPS coordinates, store names, or websites for all sampled locations. It is important to address the ethical dilemma of purchasing samples that support a trade that contributes to the overexploitation of the seahorses we are trying to protect. It is often difficult to obtain permission for collection from private sellers, and this proved true in our study as well. We were not able to get permission from private store owners to collect, necessitating the approach here to purchase samples. We do not recommend purchasing samples in further monitoring efforts, however, we needed to create baseline knowledge of species composition from TCM and ECC markets in the U.S. Secondhand reports of species composition are often vague, incorrect, or use one species as a catch-all for every individual (given the small sizes) [6,10]. We also needed to explicitly understand if sellers were following recommended size guidelines, and making direct morphological measurements was not possible from online sellers or on the spot in markets [57]. Overrepresentation of samples from a single site or shop could introduce pseudo replication to the analysis. In order to avoid this, in sites with lots/parcels containing greater than 20 individuals from the same species, we only sequenced and included a subset of individuals for the analysis (S3 Table). We also attempted random sampling by sampling once from each seller without first recognizing any species. Samples included for sequencing and analysis from stores and sites ranged from 4–21 individuals with an average of 9.85 individuals per source (S3 Table). All remaining samples that were not used for sequencing are currently being stored for future use or deposited for a museum collection. In addition, 20 individuals from a confiscated shipment (Port of New York, 2013) of unidentified *Hippocampus* spp. were provided by the U.S. Fish and Wildlife Service for identification purposes (Table 2). For additional source details see S2 Table. In both end-markets (TCM and ECC) if sellers provided more than one size grouping (i.e., small, medium or large seahorses), an equal number of individuals were selected across groupings. This study does not attempt to equate the number of species identified in our samples with the number being sold throughout the U.S., if specimens appeared morphologically distinct based on common features such as snout length, skin color patterns, and body type characters (i.e., smooth body-type, spiny body-type, coronet height, tail length, etc.), all morphotypes were purchased.

### DNA extraction, PCR, and sequencing

The majority of sample prep, morphological measurements, DNA extraction, and PCR was performed by high school students from NYC public high schools in fulfillment of Science Research courses and the Urban Barcode Project, and we have included this curriculum for future high school students in S1 File. When working with students, at the undergraduate or high school level, it is important to take extra measures to ensure quality control throughout the process. Here, all student work was performed directly under the guidance of one of the trained PhD authors of this manuscript. However, to scale this approach and include more students in the future several other quality control methods could be used including, random audits by instructors to resequence samples, have multiple students working on a small subset of the same samples to test consistency, or having pairs of students where one student’s job is to ensure proper labeling and identification throughout the protocol. Approximately a 3 x 3 mm square of muscle tissue was removed from the tail for processing. If the skin of the sample appeared to have a “glossy” appearance, it was removed to avoid contamination of unknown fixatives sometimes used to preserve specimens. Although in many cases the morphology of specimens was severely degraded, students were instructed to sample from one side of the fish so the species could be verified morphologically if necessary due to the bilateral symmetry of *Hippocampus* spp. Total genomic DNA was extracted using the DNeasy Blood and Tissue kit (Qiagen, Valencia, CA, USA). Briefly, using the standard protocol, the tissue sample was put into 180ul of digestion buffer ATL and 20ul of proteinase K at 56 degrees C for at least 2 to 3 hours with frequent vortexing during the incubation. We waited until all of the tissue was digested before proceeding.

We sequenced 267 individuals (~10 individuals per source) for either a 696-base pair (bp) segment of mitochondrial (mtDNA) gene cytb (107 individuals) or a 652bp section of gene CO1 (178 individuals). After first morphologically identifying a species, we amplified and sequenced the barcode gene (CO1 or cytb) that had more high-quality reference sequences for that given species in NCBI.

PCR amplification for CO1 and cytb took place in 25μl reactions with either 0.5μl of CO1 primers [58] (forward CO1: 10μM; dgLCO1490: GGT CAA CAA ATC ATA AAG AYA TYG G reverse CO1: 10μM; dgHCO2198: TAA ACT TCA GGG TGA CCA AAR AAY CA) or seahorse-specific cytb primers [5,52] (10μM; *shf2* TTG CAA CCG CAT TTT CTT CAG) and (10μM; *shr2* CGG AAG GTG AGT CCT CGT TG); 2.5 units of Fisher taq polymerase, 2.5μl 10X Buffer (Fisher Scientific, Inc., Pittsburgh, PA, USA), 1μl dNTP (10 mM) (Integrated DNA Technologies, Coralville, IA, USA), and 1μl of genomic DNA. PCR thermal cycling conditions for CO1 were: 5 min at 95°C; 35 cycles of 30s at 95°C; 1 min at 50°C; 1 min at 72°C; and a final 10 min at 72°C. The amplification protocol for PCR reactions for cytb was: 94°C for 5 min; 35 cycles of 94°C for 30 s; annealing temperature 52°C for 1 min; 72°C for 1 min; final extension 72°C for 10 min; final rest at 4°C. Possibly due to DNA degradation, some samples yielded lower quality DNA. If initial PCR amplification produced faint bands using the aforementioned protocols, PCR amplification was increased to 39 cycles, with annealing temperatures decreased by 2 degrees (48°C for CO1 and 50°C for cytb) yielding successful PCR amplification. The latter PCR modification was only used when first round PCR failed because while reducing the annealing temperature can increase product, it can also produce non-specific binding. PCR products were visualized on 1.5% agarose gels stained with SYBR^®^Safe (Thermo Fisher Scientific, Waltham, MA, USA.). PCR samples were purified with Agencourt AMPure XP (New England BioLabs, Ipswich, MA, USA), sequenced with BigDye version 3.1 chemistry, and sequenced using an ABI3730 genetic analyzer (Applied Biosystems, Inc., Foster City, CA, USA). All sequences were aligned using Geneious Pro v.6 [59].

### Specimen identification

For specimen identification, species were identified based on sequence similarity using the BLASTn algorithm against the GenBank nt database and further validated using neighbor-joining phylogenies. To assign a species status to a given specimen based on genetic similarity, the mean and range of intraspecific and interspecific sequence divergence for *Hippocampus* spp. was calculated based on pairwise K2P distances using MEGA 5.2 [60] from 180 available CO1 reference sequences (>600bp; 24 reference species, S4 Table) and 580 cytb sequences (>600bp; 26 reference species, S5 Table). Both datasets include all species commonly reported in the dried seahorse trade [5]. Neighbor-joining phylogenetic trees [61] were generated in Geneious v.6.0 [59] using the HKY model of sequence evolution. Majority-rule consensus trees (50%) were constructed from 1,000 bootstrap replicates per phylogeny (S1 Fig).

### Total height per individual

**Fig 2:**
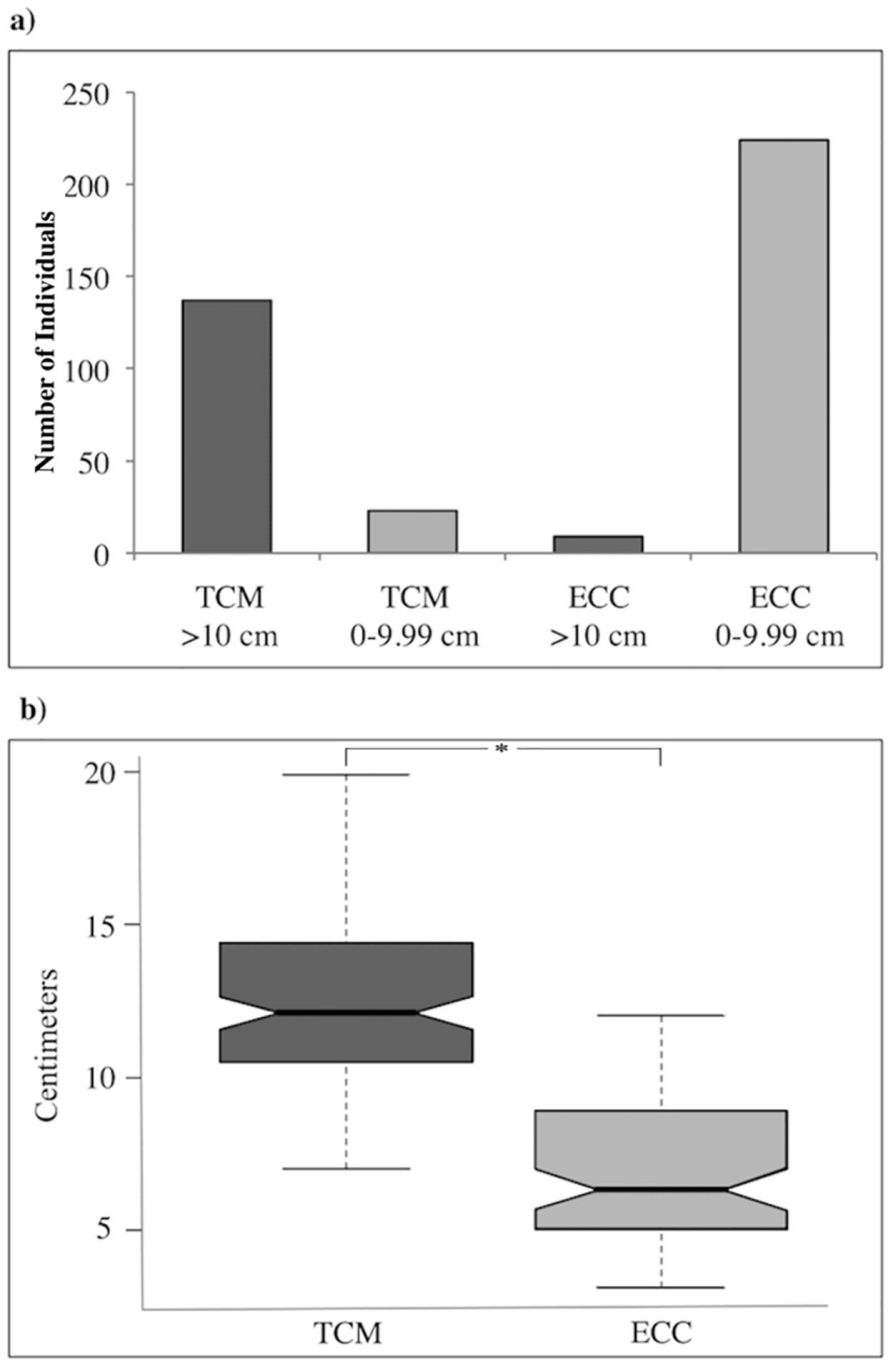
Size range of seahorse samples per market. (a) Specimens were divided into two categories >10-cm and <10-cm for each market (TCM and ECC). Histogram values on y-axis show number of individuals sampled per category. ECC values do not include the dwarf seahorse *H*. *zosterae*. (b) Boxplots illustrating the distribution of size variance across individuals per category between all TCM and ECC samples combined. The notches represent the 95% CI around the median for each group and * = *T-test P* < 0.0001.

Total height was measured for each seahorse to examine if the current CITES 10 cm minimum size limit recommendations were met, as well as to determine the proportion of sizes in each U.S. end-market. The 10 cm minimum size limit allows for any variation in sample sized caused by the drying process because the majority of seahorses reach sexual maturity at 7 cm [4]. Total height measurement protocols followed recommendations of [5]. Measurements were included for all specimens except those from *H*. *zosterae*, with adults typically ranging in size from 1.6–3.8 cm [62]. Specimens were measured laterally from the distance of the prehensile tail (posterior) to the top of the coronet (anterior) using a flexible measuring device to account for variations in shape as illustrated in (S1 File). Using R v.4 (R-project.org) the size in centimeters was compared between TCM and ECC samples (Fig 2). To test the significance of the size difference comparison between groups, a Student’s t-test was conducted and boxplots were generated with the notch function that represents the 95% confidence interval (CI) around the median.

### Calculating the minimum number of seahorses per kilogram for U.S. import estimates

To estimate the total number of individuals being imported into the U.S., we utilized reported CITES records of all Syngnathidae imported from 2004–2020. All data is publicly available from the CITES trade database (trade.cites.org). When units are available most dried seahorses are reported in kilograms (kg), grams (g), or number of specimens. To derive an estimate of the number of seahorses per kg from our TCM samples, 133 whole individuals were weighed. We calculated a mean weight of 2.78 g/seahorse (individuals ranged from 1.03–5.25 g/seahorse) equivalent to 359.7 individuals per kg. We included estimates for mean individuals/per kg from [27,28] (Table 1) calculated from counting number of seahorses per kilogram across 13 countries across Asia and surveys from fishermen and sellers, and this generated a global value of 370.4 individuals/kg.

**Table 1:** Estimates of average dry seahorse weight.

Region/Country	Estimated seahorse dry weight (g/seahorse)	Individuals/kg.	Reference
Australia	3.00	333.33	(Martin-Smith, 2006)
Latin America (Atlantic)	2.42	413.22	(Baum and Vincent, 2005)
Latin American (Pacific)	3.51	284.90	(Baum and Vincent, 2005)
Malaysia	3.18	314.47	(Perry et al., 2010)
Thailand	3.13	319.49	(Perry et al., 2010)
Thailand	3.30	303.03	(Vincent, 1996)
Philippines	3.33	300.30	(Pajaro and Vincent, 2015)
Philippines	1.38	724.64	(Vincent, 1996)
India	1.50	666.67	(Vincent, 1996)
Indonesia	2.00	500.00	(Vincent, 1996)
Vietnam	2.86	349.65	(Vincent, 1996)
U.S. TCM samples	2.78	359.71	(This study)
**Global Estimate**		**370.4**	

Data (g/seahorse) per region/country complied from [28].

**Fig 3:**
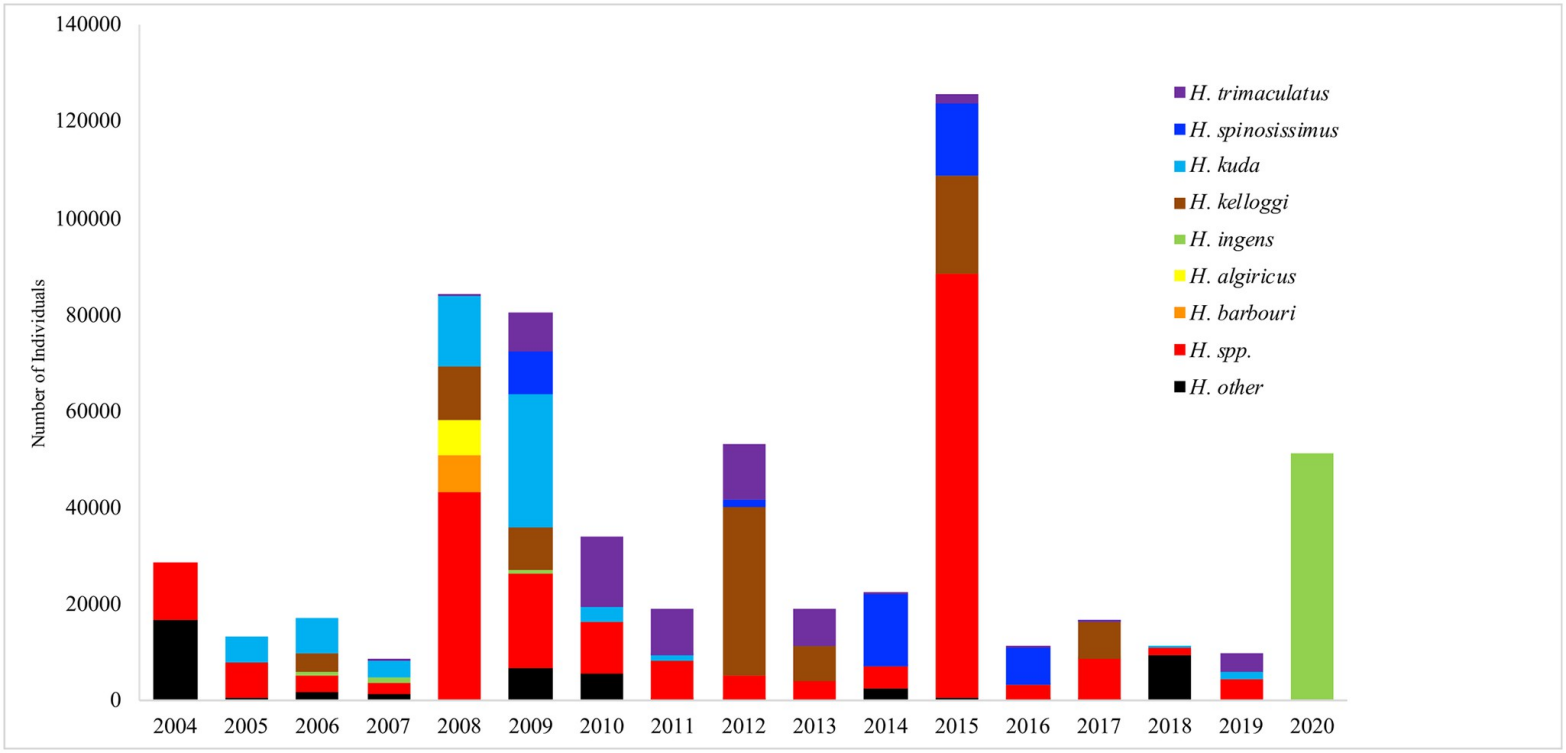
Estimates of import abundance per year derived from CITES import records. Estimates of individuals per year per species or group (i.e., *Hippocampus* spp. and “*Hippocampus* other”) with legend colors representing each year that CITES data was publicly available (2004–2020, more data may be released as there is a lag between the year and when data is posted). estimates are based on a mean “global” weight of 370.4 individuals/kilogram. Raw data reported in S6 Table.

Based on this value, we estimated the total number of individuals per species imported into the U.S. starting with 2004, the year *Hippocampus* was added to CITES listing. Estimates of the total number of individuals imported into the U.S. were calculated for each species in this study. All records labeled as *Hippocampus* spp. were combined, and all other species not identified in our samples were combined as a single estimate, “*Hippocampus* other” (Fig 3). We only included data that specified units (i.e., kilograms, grams or specimens) and excluded records where units were not reported, or were recorded as “live,” or as “derivatives”. This latter designation may be medicinal products that contain seahorse material (i.e., pills and capsules), though the content weight of *Hippocampus* is unknown.

## Results

### Specimen identification

The results of this study show a substantial difference in the species composition between the specimens from ECC and TCM markets. For those samples that were sequenced and quality filtered, specimen sequence length for TCM and ECC samples ranged from 615-652bp (CO1) and 658-696bp (cytb). Generated sequences are available on GenBank (Accession numbers: cytb; KT591945—KT59205 and CO1; KT592052—KT592229).

The intraspecific and interspecific pairwise genetic distances for *Hippocampus* were calculated for both barcode genes. The interspecific mean K2P distance for CO1 was 11.49% (range: 1.0–17.2%), and a mean intraspecific distance of 0.76% (range: 0.0–3.33%). For Cytb, the intraspecific mean K2P distance was 0.66% (Range: 0.0–1.67%), and the mean interspecific distance was 15.3% (range 1.2% - 24.0%). The 50% majority-rule neighbor-joining phylogenies further supported sequence similarity identification of specimens, (S1 Fig).

We assigned species with the Blastn algorithm based on the lowest interspecific genetic distance (<1%; CO1 and <1.2%; cytb), with the exception of the dwarf seahorse, *H*. *zosterae*, of which the sequence similarity ranged from 98.2–100%. For the remaining samples, sequence similarity ranged from 99.1–100.0% (S3 Table). This allowed for the clear identification of all but three specimens that could not be confidently distinguished between the trans-Atlantic sister-species *Hippocampus reidi* and *Hippocampus algiricus* (<0.6% Blastn genetic distance between reference sequences), but showed a 99.6–99.9% sequence similarity to *H*. *algiricus*. In addition, the neighbor-joining tree placed these specimens in a monophyletic clade with *H*. *algiricus* reference sequences (S1 Fig). Therefore, we putatively designated these specimens as *H*. *algiricus*. Additional details including genetic distance to reference sequences, and phylogenetic trees can be found in S3 Table and S1 Fig. We also report any additional species that fell with 98% similarity to any of our sequences. The total numbers of individuals per species/per market are summarized in Table 2.

**Table 2:** Species market composition.

Species	2010 TCM Philadelphia	2009–2013 TCM: New York	2011–2014 ecommerce stores	2013 coastal stores: Texas	2013 Confiscated shipment	Sanders et al. (2007) TCM: California	Sanders et al. (2007) curio: California	IFAW Report (2000) TCM	Total across reports
*H*. *ingens* Girard, 1859	8	28	0	0	0	29	0	32	97
*H*. *kelloggi* Jordan & Snyder, 1902	4	6	0	0	0	0	0	21	31
*H*. *trimaculatus* Leach, 1814	3	48	0	11	15	3	1	58	139
*H*. *spinosissimus* Weber, 1913	10	28	0	2	5	6	0	42	93
*H*. *comes* Cantor, 1850	0	6	0	0	0	4	0	11	21
*H*. *kuda* Bleeker, 1852	0	23	177	0	0	0	0	13	213
*H*. *algiricus* Kaup, 1856	0	3	0	0	0	0	0	5	8
*H*. *zosterae* Jordan & Gilbert, 1882	0	0	123	17	0	0	0	0	140
*H*. *barbouri* Jordan & Richardson, 1908	0	1	0	14	0	0	9	5	29
*H. spp.	0	0	0	0	0	1	0	19	20
***Total***	**N = 25**	**N = 143**	**N = 300**	**N = 39**	**N = 20**	**N = 46**	**N = 10**	**N = 206**	**N = 792**

Species composition of samples collected for this and two previous studies on the U.S. dried seahorse market. Row *H. spp. is composed of 20 individuals: unidentified *H*. spp. (n = 11), *H*. *hippocampus* (Linnaeus 1758) (n = 1), *H*. *angustus* (n = 4), *H*. *erectus* (n = 3), *H*. *whitei* (n = 1), and *H*. *fuscus* (n = 1).

Based on these results five species were identified in the ECC market. The most abundant species were identified as mostly juvenile specimens (<10 cm) of *H*. *kuda* (51.5% of ECC samples) and the domestic dwarf seahorse *H*. *zosterae* (40.7% of ECC samples). In contrast, eight species (including the putative *H*. *algiricus* specimens) were identified from TCM samples with *H*. *trimaculatus*, *H*. *spinosissimus*, and *H*. *ingens* totaling 75.0% of TCM samples (Table 2). In addition, individuals from the confiscated shipment into the Port of New York were identified as *H*. *trimaculatus* (75%) and *H*. *spinosissimus* (25%). Fig 1 illustrates the distributions of each species identified in this study and potential geographic range of the U.S. import seahorse fishery.

### Size of individuals between markets

A significant difference was found between the average size of specimens available for purchase between TCM and ECC markets (T-test; p-value < 0.001, Fig 2b). TCM samples >10-cm made up 85.7% of the total collected (mean: 12.9 cm, range: 10–19.9 cm). In stark contrast, only 4.8% of 204 ECC samples (excluding the dwarf species *H*. *zosterae*) were >10-cm (range: 10.0–12.1 cm).

### Import abundance estimates

Reported CITES imports of dried seahorses totaled approximately 972,446 individuals. Reported import abundance is presented for each species identified in this study (Fig 3, *H*. *comes* was not reported on CITES), and also includes undesignated species imports (*Hippocampus* spp.) and species not found in our study (*Hippocampus* other). The species *H*. *trimaculatus*, *H*. *kuda* and *H*. *kelloggi* are the most reported species, while *Hippocampus barbourin*, *H*. *ingens*, and *H*. *spinosissimus* appear to make up only a small portion of reported imports.

## Discussion

While only a glimpse into the overall U.S. seahorse trade, this is one of the only studies to extensively sample dried seahorses sold as curios through ecommerce (ECC) to conduct a comparison with the traditional Chinese medicine (TCM) market. This required the purchasing of samples to create this knowledge base, but now that we have species composition of the ECC market, moving forward we would recommend to avoid purchasing samples that may support this trade. Our results show a significant difference in the size of individuals and composition of species between markets. Compared to TCM, we found a higher proportion of the domestic dwarf seahorse *H*. *zosterae* among ECC samples. We found a large species overlap between our sampled confiscated shipment and TCM/ECC markets. The majority of confiscations usually lack species designations because they are rarely investigated by experts, so molecular identification of samples from confiscated shipments can shed light on unreported imports. The large similarity in species composition between markets and confiscated shipments suggests these shipments may be a source for markets and sampling confiscated shipments may serve as a proxy for what is sold in TCM/ECC markets. However, the one shipment studied here is not enough to confirm its use as a proxy and will require further sampling of multiple shipments over time to establish this link. Also, proxy status will not be true indefinitely. Many factors including the frequency of confiscation and changing dynamics in how samples are obtained can change this proxy status requiring a new investigation into TCM and ECC markets in the future.

### Contrasts between TCM and ECC markets and species exploitation trends

Two previous studies examining the dried TCM seahorse species composition in the U.S. focused on samples collected in California [8] (n = 46), and from Chinatowns in Boston, MA., New York, NY., Washington, DC., Los Angeles, Oakland, and San Francisco, CA (n = 200) by the International Fund for Animal Welfare (IFAW 2000) [6,9]. We found a similar composition of our TCM samples with these previous studies. The majority (75.0%) of the TCM samples identified in this study were composed of *H*. *trimaculatus*, *H*. *spinosissimus* and *H*. *ingens*. These were also the most abundant species in the IFAW Report (66% of specimens), while nearly two-thirds of the California samples were identified as *H*. *ingens* (Table 2).

All three of these species are considered vulnerable on the IUCN Red List due to their abundance in the global TCM trade and prevalence as bycatch [7,63,64]. Both *H*. *trimaculatus* and *H*. *spinosissimus* populations have declined recently by as much as 30% across several countries as reported by local fishers and traders [4,17,64,65], while heavy fishing pressure has caused a significant decline in *H*. *ingens* [7] with Latin America reporting 1.5 tons of dried specimen exports (approximately 500,000 individuals) in 2004 alone [8].

In contrast to the specimen composition of the TCM samples, *H*. *kuda* and *H*. *zosterae* totaled 93.2% of all ECC samples. The Indo-Pacific *H*. *kuda* was identified in both TCM and ECC samples, and is heavily exploited in the international TCM trade with many populations considered threatened due to habitat degradation [5,26,66]. The dwarf seahorse *H*. *zosterae*, native to North and Central America was not found in TCM samples, and previous research has not listed it as part of the dried seahorse trade [5,6]. This may be because most previous research has focused on Indo-Pacific species. Also, in our study, samples were collected at one timepoint from each seller, so the discrepancies in species composition between TCM and ECC may be due to the simple fact that the originating import shipments came from different geographies.

### Exploitation of the domestic dwarf seahorse *Hippocampus zosterae*

The domestic seahorse, *Hippocampus zosterae*, may be an important species to monitor going forward, given the high occurrence of it in our dataset and being an easily accessible domestic sample. In 2012, under the “Animals and Wildlife Product Policy,” eBay agreed that the sale of dried imported seahorse specimens would no longer be allowed for sale on their site (pages.ebay.com/help/policies/wildlife), and as of 2020 all seahorses were banned from being sold on eBay and etsy. However, seahorses are still able to be sold on other independent ecommerce sites within the US and as a domestic species, *H*. *zosterae* may be an easily accessible animal for collection. *H*. *zosterae* is also a popular species in the global aquarium market [14,27,67], and recorded catch records from Florida show that it occupied the second most abundantly exported species of the top 10 aquarium fishes sold for export [67]. To our knowledge, catch landings for dried *H*. *zosterae* specimens are unreported, yet the high numbers found in our samples indicate that this species is easily purchased from U.S. retailers through ecommerce as curio products. *H*. *zosterae*’s small size and low fecundity may make it particularly vulnerable to overfishing. For example, most species of *Hippocampus* release approximately 100–300 young [5,68], while *H*. *zosterae* may produce as few as five young per brood [69]. (The most recent IUCN Red List has *H*. *zosterae* as Least Concern and most populations across its range are stable or increasing as determined by NOAA in 2020).

### Market variability in following CITES size limit recommendations

While some seahorses are directly targeted, up to 95% of seahorses in the international trade are still collected by non-selective fishing bycatch; most prominently in shrimp trawls [9,19,29]. In the last decade, research concluded that 15 of the 16 most traded seahorse species would have the potential to reproduce before harvest if a 10 cm minimum height limit was implemented [19]. This research prompted the CITES Animals Committee and all signatory nations to agree to the 10 cm minimum size limit recommendation to create a more sustainable seahorse trade [6; cites.org].

Three of the species identified in our samples—*Hippocampus kelloggi*, *H*. *ingens* and *H*. *kuda* may reach reproduction after 10 cm in height (S1 Table). Most TCM samples were >10 cm, in contrast to the high abundance of individuals <10 cm in the ECC market, composed primarily of *H*. *kuda* and *H*. *zosterae* (Fig 2). ECC markets, made up of local U.S. online sellers, may be dominated by domestic *H*. *zosterae* due to easy access of abundant populations in the Gulf of Mexico. The international trade of seahorses is allowed if sourced sustainably and legally, but is voluntarily regulated by the CITES signatory nations and implemented based on individual country management efforts. CITES recommends bans from several traditional large seahorse export countries, like Thailand, the Philippines, and China [29], however, many exports first go to Hong Kong (unaffiliated with CITES regulations) before being traded globally [29]. Therefore, it is difficult to know if seahorse imports are following regulations or even quantify the impact the CITES minimum size recommendation has had on what size seahorses are being harvested from fisheries globally. We did not find any unexpected species in our study and without any caps on import numbers, trade will likely continue. It is important to monitor the trade, however, as seahorses are vulnerable to overexploitation due to their life history traits and prevalence as by-catch in other fisheries.

### U.S. import abundance based on CITES records, unreported imports, and confiscations

CITES data has known challenges to analyze trade in seahorses [9], nevertheless, the dataset is unparalleled for investigating listed species including seahorses [28]. Because of the public availability of all CITES records, we were able to use these data to estimate the number of individuals imported into the U.S. from 2004–2018. Our results indicate that 972,446 dried individuals were imported into the U.S. from 2004–2018 (approximately 2,625 kg) based on a mean estimate of 370.4 individuals per kg (Table 1). Previous research has estimated the number of individuals per kilogram and showed a dramatic range based on region, from 285 to 724 seahorses/kilogram) [28,70,71]. Therefore, if the upper and lower estimates are used instead of our global mean value, the number of individuals imported into the U.S. would range from 747,862 to 1,902,172 individuals. We emphasize that these results are only an approximation, due to the high variance. Furthermore, this estimate is conservative due to the exclusion of any ambiguous import records where the weight of the import was unspecified. Despite this uncertainty, this estimate can be used as a baseline of the relative abundance of dried seahorses being sold in the U.S. since the establishment of CITES listing of the genus *Hippocampus*.

The analysis of available CITES records also shows that the total number of individuals and types of species imported into the U.S. varies dramatically from year to year (Fig 3). These import records present a discrepancy with the U.S. TCM markets where *H*. *spinosissimus* and *H*. *ingens* were two of the three most commonly found species (Table 2 and Fig 3). Among all the identified samples in this study, only the species *Hippocampus comes* remained unreported to CITES.

Unreported shipments that are confiscated often lack species designations and lead to further discrepancies in species-specific import records. For example, of the 827 available import records in the CITES database 39.8% (329 records) were confiscated shipments, with the vast majority (85.1%, 280 records) recorded as *Hippocampus* spp. Of confiscated imports, 58.4% of (192 records) were dried specimens, 31.9% (105 records) “derivatives,” and 9.7% (32 records) “live” specimens. Of the confiscated imports of dried specimens only 8.2% (16 records) had a designated species status.

As an additional component of our research we sequenced individuals from a confiscated shipment into the Port of New York in 2013 (*Hippocampus* spp.) to identify the species composition of this shipment. We identified *H*. *trimaculatus* and *H*. *spinosissimus* (Table 2) which were also the two most abundant species found in our TCM samples. Using confiscated samples to support research on imports/exports is important and government agencies are now utilizing molecular labs to some degree to identify unidentified wildlife and validate food products [72]. Much of the confiscated shipments, however, remain unidentified to the species level making it difficult to have an accurate view of the wildlife trade. This is the major part of the seahorse trade missing verifiable numbers. More shipments should be sent to forensics labs for identification especially given the advancement in portable technology and protocols [45,48], and the potential for confiscated shipments to serve as proxies for the TCM and ECC trade. The general utility of wildlife identification by non-experts, including students, citizen scientists, and natural resource professionals using DNA barcoding demonstrates the potential for this method to work in tandem with CITES to retroactively validate the species composition of confiscated imports that lack species designations or verify import documentation.

## Conclusions

Our results show that the most striking disparities between U.S. TCM and ECC markets are significant differences in species composition and size of specimens. Size comparisons show the majority of specimens sold as curio were collected before reaching the size of first reproduction, a potential consequence of seahorses often getting collected as bycatch [73]. A continued effort needs to be made by CITES signatory nations to emphasize the long-term benefit of a sustainable harvest to fishers to reduce non-target bycatch [13,74]. The finding of domestic dwarf seahorse *H*. *zosterae* being sold in the ECC market indicates that this species is no longer only exploited for the live aquarium trade. Our results also demonstrate that molecular identification of confiscated imports which lack species designations could serve as a proxy to supplement seahorse trade information. Lastly, we are contributing to prevalent and robust DNA barcoding curriculum to continue helping students conduct molecular biology research of food and wildlife products and gain experience with authentic research experiences in the classroom.

## Supporting information

S1 Fig CO1 and Cytb neighbor-joining 50% majority consensus phylogenetic trees. Neighbor-joining 50% majority consensus tree visualized in FigTree v1.3.1 (Rambaut, 2012) generated from 1,000 bootstrap replicates in Geneious v6.0 (Drummond et al. 2011). Node support presented for each clade with specimens collected for this study. Reference sequences for both cytochrome b (cytb) and cytochrome oxidase subunit 1 (CO1) were selected from available references sequences used to generate intraspecific and interspecific K2P genetic distances. References sequences are designated by species name.
(TIFF)

S1 Table Reproductive traits for the relevant species found in this paper. Life history traits relevant to survival, demographic history, and susceptibility to overexploitation for all seahorse species found in this study including relevant citations.
(TIFF)

S2 Table Collection site metadata. Information on collection sites and store type, specimens, and descriptions. Specified descriptions refer to terms used by sellers.
(TIFF)

S3 Table Information on % genetic distance similarity of specimens collected to GenBank reference sequences. Additional information includes sequence length, purchase location (See S2 Table), and barcode gene sequenced for identification purposes.
(TIFF)

S4 Table COI_Summary of the results from the Kimura 2-parameter model (K2P) estimation across samples and reference species. The average K2P for each species (Minimum, Maximum) are reported in each cell. Species in Bold and with a ’*’ represent species with only one reference sequence and do not report min or max.
(TIFF)

S5 Table CytB_Summary of the results from the Kimura 2-parameter model (K2P) estimation across samples and reference species. The average K2P for each species (Minimum, Maximum) are reported in each cell. Species in Bold and with a ’*’ represent species with only one reference sequence and do not report min or max.
(TIFF)

S6 Table Gross import abundance into the U.S. per year from CITES import records. The data shows gross imports of the genus *Hippocampus* into the United states of America between the years 2004 and 2022 (the most recent data available) from CITES records, https://trade.cites.org/. Counts show reported bodies and/or specimens. If the import record was reported in kilograms, it was converted to individuals based on 370.4 individuals/kilogram. *Hippocampus spp*. represents reported records without species classification. Any *Hippocampus* species reported but not found in this study are grouped into the category, “*Hippocampus other”*.
(PDF)

S1 File Curriculum guide. Citizen science and student led wildlife trade investigations.
(TIFF)
