# Supplementary figures and images for: The United States dried seahorse trade: A comparison of traditional Chinese medicine and ecommerce-curio markets using molecular identification
Source: PLoS One. 2023 Oct 3;18(10):e0291874. doi: 10.1371/journal.pone.0291874 (PMC10547177; doi:10.1371/journal.pone.0291874)

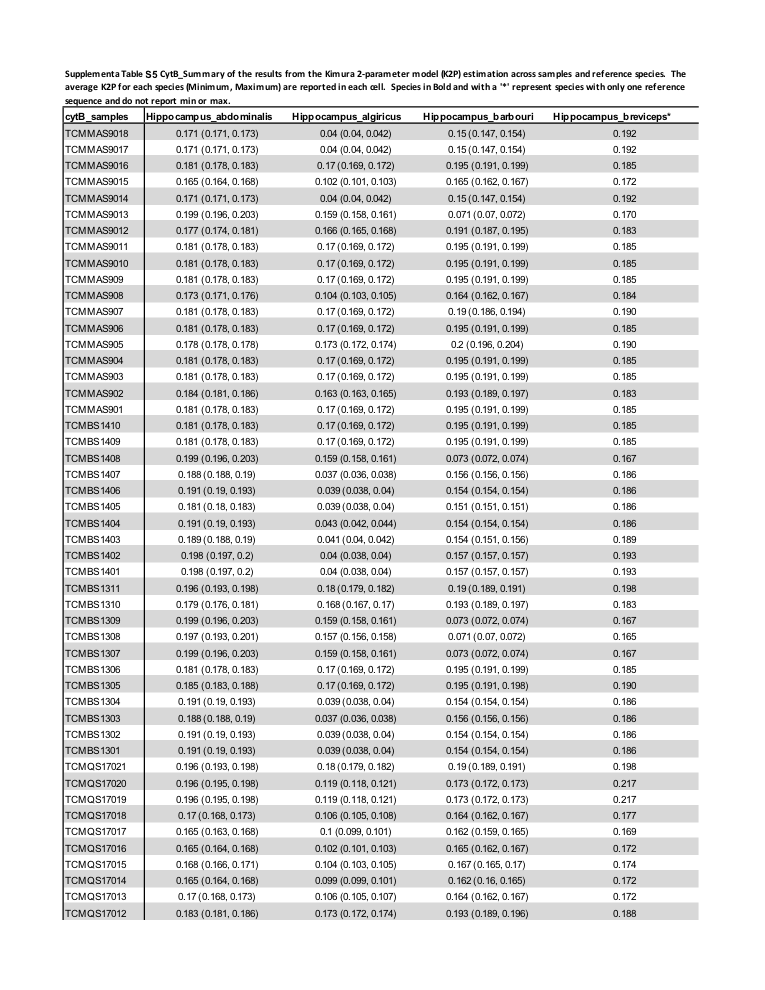

Supplement: S5 Table — The average K2P for each species (Minimum, Maximum) are reported in each cell. Species in Bold and with a ’*’ represent species with only one reference sequence and do not report min or max. (TIFF) [file pone.0291874.s006.tiff]

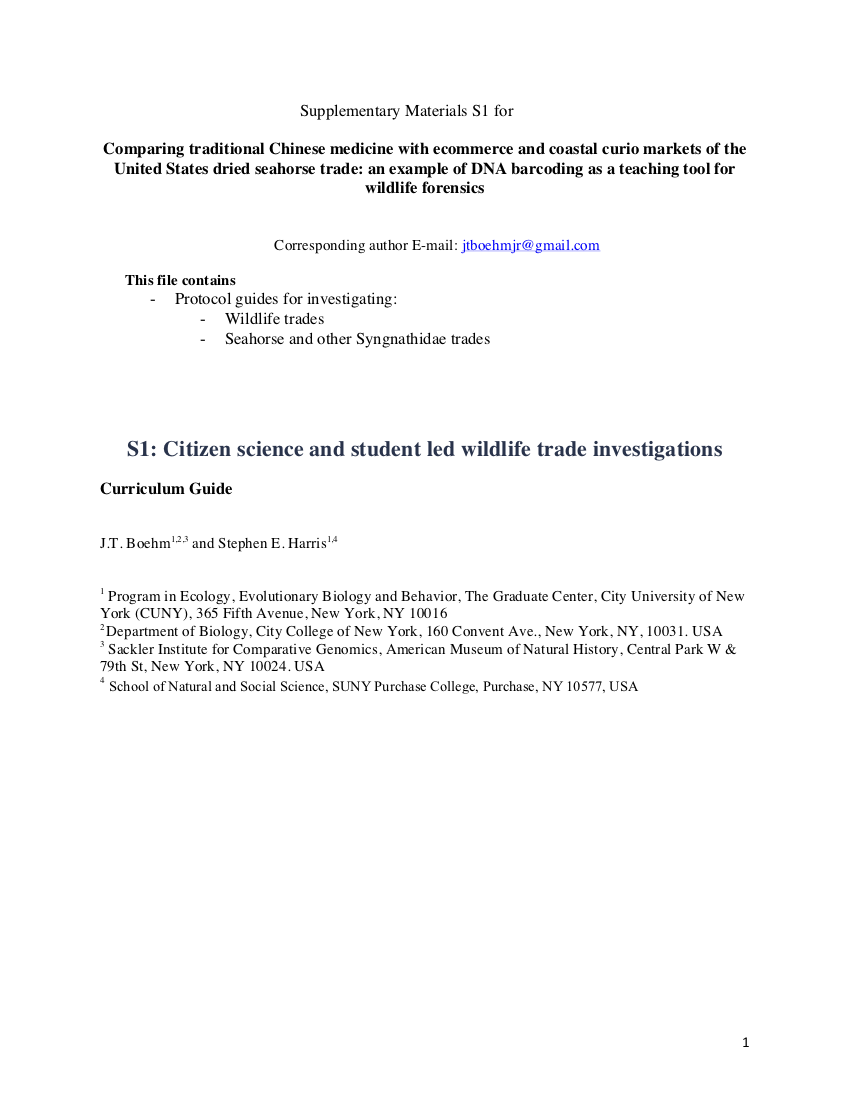

Supplement: S1 File — Citizen science and student led wildlife trade investigations. (TIFF) [file pone.0291874.s008.tiff]
