# Supplementary material for: The United States dried seahorse trade: A comparison of traditional Chinese medicine and ecommerce-curio markets using molecular identification
Source: PLoS One. 2023 Oct 3;18(10):e0291874. doi: 10.1371/journal.pone.0291874 (PMC10547177; doi:10.1371/journal.pone.0291874)
Supplement: S6 Table — The data shows gross imports of the genus Hippocampus into the United states of America between the years 2004 and 2022 (the most recent data available) from CITES records, https://trade.cites.org/. Counts show reported bodies and/or specimens. If the import record was reported in kilograms, it was converted to individuals based on 370.4 individuals/kilogram. Hippocampus spp. represents reported records without species classification. Any Hippocampus species reported but not found in this study are grouped into the category, “Hippocampus other”. (PDF) [file pone.0291874.s007.pdf]

Supplemental Table S6 Gross import abundance into the U.S. per year from CITES import records. The data shows gross imports of the genus *Hippocampus* into the United states of America between the years 2004 and 2022 (the most recent data available) from CITES records, https://trade.cites.org/. Counts show reported bodies and/or specimens. If the import record was reported in kilograms, it was converted to individuals based on 370.4 individuals/kilogram. *Hippocampus spp.* represents reported records without species classification. Any *Hippocampus* species reported but not found in this study are grouped into the category, "*Hippocampus other*".

	2004	2005	2006	2007	2008	2009	2010	2011	2012	2013	2014	2015	2016	2017	2018	2019	2020	2021	2022	Grand Total
H. trimaculatus				151	23	8161.2752	14816	9339	11566.104	7430	320	1889	131	3		3787				57616.3792
H. spinosissimus						8610.2784		1	1552.272	24	14885	14816	7408	5						47301.5504
H. kuda		5160	7440	3400	14612	27900	3050	1203			15	6	148	15	401.4	1852				65202.4
H. kelloggi			3873		11112	8741.0696			35188	7409		20372		7413						94108.0696
H. ingens			740.8	1069.8		667							3	3			51269			53752.6
H. algiricus					7408									2						7410
H. barbouri			30		7410		20					10	8	4						7482
H. spp.	11644.4	7402.3424	3556.3264	2418.0088	43172.4704	19642.0492	10792.9088	8163.1608	4845.7712	3768.958	4721.44	88001.8609	2994.9856	8556.0928	1713.2032	4110.4				225504.378
H. other	16668	376.4	1481.6	1174.8	12	6571.4656	5320.1536	37	4	160	2244.4	525	112	98	9113.6					43898.4192
Grand Total	28312.4	12938.7424	17121.7264	8213.6088	83749.4704	80293.138	33999.0624	18743.1608	53156.1472	18791.958	22185.84	125619.861	10804.9856	16099.0928	11228.2032	9749.4	51269	0	0	602275.797
